# A Consensus Microsatellite-Based Linkage Map for the Hermaphroditic Bay Scallop (*Argopecten irradians*) and Its Application in Size-Related QTL Analysis

**DOI:** 10.1371/journal.pone.0046926

**Published:** 2012-10-16

**Authors:** Hongjun Li, Xiao Liu, Guofan Zhang

**Affiliations:** Institute of Oceanology, Chinese Academy of Sciences, Qingdao, China; Biomedical Research Institute, United States of America

## Abstract

Bay scallop (*Argopecten irradians*) is one of the most economically important aquaculture species in China. In this study, we constructed a consensus microsatellite-based genetic linkage map with a mapping panel containing two hybrid backcross-like families involving two subspecies of bay scallop, *A. i. irradians* and *A. i. concentricus*. One hundred sixty-one microsatellite and one phenotypic (shell color) markers were mapped to 16 linkage groups (LGs), which corresponds to the haploid chromosome number of bay scallop. The sex-specific map was 779.2 cM and 781.6 cM long in female and male, respectively, whereas the sex-averaged map spanned 849.3 cM. The average resolution of integrated map was 5.9 cM/locus and the estimated coverage was 81.3%. The proportion of distorted markers occurred more in the hybrid parents, suggesting that the segregation distortion was possibly resulted from heterospecific interaction between genomes of two subspecies of bay scallop. The overall female-to-male recombination rate was 1.13∶1 across all linked markers in common to both parents, and considerable differences in recombination also existed among different parents in both families. Four size-related traits, including shell length (SL), shell height (SH), shell width (SW) and total weight (TW) were measured for quantitative trait loci (QTL) analysis. Three significant and six suggestive QTL were detected on five LGs. Among the three significant QTL, two (*qSW-10* and *qTW-10*, controlling SW and TW, respectively) were mapped on the same region near marker *AiAD121* on LG10 and explained 20.5% and 27.7% of the phenotypic variance, while the third (*qSH-7*, controlling SH) was located on LG7 and accounted for 15.8% of the phenotypic variance. Six suggestive QTL were detected on four different LGs. The linkage map and size-related QTL obtained in this study may facilitate marker-assisted selection (MAS) in bay scallop.

## Introduction

Linkage maps have become important genetic tools which provide detailed information on genotype-phenotype relationships [1]. Quantitative trait loci (QTL) analysis based on linkage map reveals the genetic basis and inheritance patterns of economically important traits, which is essential for marker-assisted selection (MAS) in future breeding programs. Genetic linkage maps have been published for some economically important aquaculture species such as the Asian seabass (*Lates calcarifer*) [2], the Atlantic salmon (*Salmo salar*) [3], the Japanese flounder (*Paralichthys olivaceus*) [4], the rainbow trout (*Oncorhynchus mykiss*) [5], the Pacific white shrimp (*Litopenaeus vannamei*) [6] and the tilapia (*Oreochromis* spp.) [7]. Although marine bivalves represent a major portion in the aquaculture industry, only a few linkage maps have been constructed mainly using amplified fragment length polymorphisms (AFLPs). These AFLP-based linkage maps include the oysters (*Crassostrea virginica* [8], *Crassostrea gigas* [9] and *Ostrea edulis* [10]), the pearl oysters (*Pinctada martensii* [11] and *Pinctada fucata* [12]), the mussel *Mytilus edulis* [13] and the scallops (*Chlamys farreri* [14], [15] and *Argopecten irradians* [16], [17]).

Many economically important traits of aquaculture species are continuous quantitative in phenotype, which are controlled by multiple loci across the whole genome. The identification and mapping of QTL controlling economic traits represent an important approach towards determining the molecular genetic basis of some complex traits, especially the size-related QTL in mollusks. Although QTL analysis in mollusk may have been limited by the lack of inbred lines, F_2_ populations and backcross families, the high fecundity and high levels of polymorphisms among different populations of mollusks may facilitate QTL mapping in these animals [18]. A few QTL controlling growth and disease resistance traits have been located on some linkage maps in marine bivalve such as the oysters [19]–[21] and the scallops [16], [18], which will be useful for MAS in these aquaculture species.

Bay scallop (*Argopecten irradians*) is a simultaneous hermaphroditic marine bivalve, with uncontinuous distribution along the eastern coast of United States and Gulf of Mexico. It can be further divided into three geographical subspecies: *A. i. irradians*, *A. i. concentricus* and *A. i. amphicostus* [22]. Since early 1980s, two subspecies, *A. i. irradians* and *A. i. concentricus* have been successively introduced to China and quickly gained popularity in Northern and Southern China respectively [23]. The annual production of bay scallops in China increased steadily from over 200,000 tons in 1990s [24] to 600,000 tons in 2007 [25]. Genetic improvement has contributed, at least partially, to the great success of bay scallop aquaculture in China [25]. The efficiency of genetic improvement depends largely on the availability of genetic information such as linkage maps. Although we have developed sex-specific linkage maps for bay scallop mainly using AFLP markers [16], such dominant markers may not be easily shared among different mapping populations established by different laboratories. Among the currently available molecular markers, microsatellite markers are the most popular choice for linkage mapping. Microsatellites are short (1–6 bp) repetitive DNA sequences, which are highly abundant and evenly distributed throughout genomes [26], [27]. As codominant markers, microsatellite data may transfer well among different mapping populations and will be necessary for future QTL studies among different studies. In this study, we present a consensus genetic map using microsatellite markers for bay scallop, which may provide a scaffold to enable integration with novel markers.

## Materials and Methods

### Mapping families

**Figure 1:**
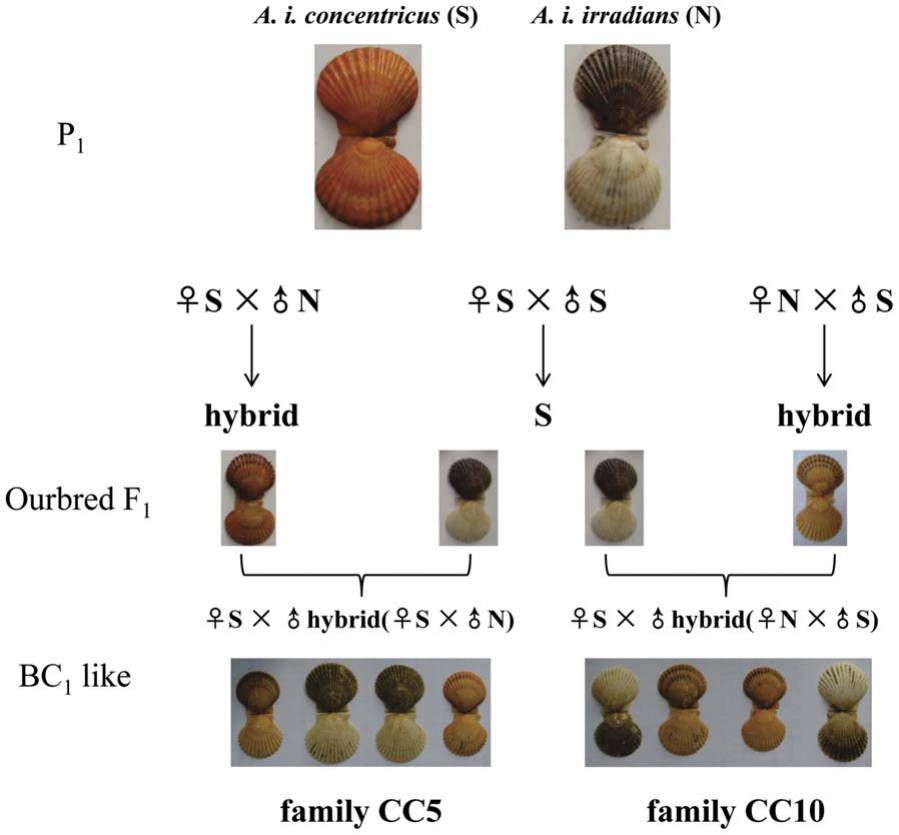
Experimental mating scheme employed to generate the two hybrid backcross-like mapping families (CC5 and CC10) and segregation of shell color in offspring.

The mapping panel used in this study included two hybrid backcross-like families constructed with two geographical subspecies of bay scallop: the northern bay scallop *A. i. irradians* (denoted as N) and the southern bay scallop *A. i. concentricus* (denoted as S). The N stock was initially introduced from Massachusetts and Virginia (USA) in 1998 and 1999 [28], and has been cultured mainly in Bohai Bay in Northern China. The brood stocks of N stock used in this study have been selected for orange shell color for 3 generations [29]. The brood stocks of S stock, whose shell color is black on the left valve and white on the right, were obtained from a hatchery stock cultivated in Guangdong province since its initial introduction from Florida (USA) in 1991 [30]. Two hybrid families (♀N×♂S and ♀S×♂N) were produced by crossing the N and S stocks in a hatchery in Qingdao. Two backcross families (Figure 1) were constructed for mapping: (1) CC5: a crossbred family between a ♂ hybrid (♀S×♂N) and a ♀S, denoted as ♀S×♂(S×N); (2) CC10: a crossbred family between a ♂ hybrid (♀N×♂S) and a ♀S, denoted as ♀S×♂(N×S). As these crossbred families were not derived from inbred lines of Northern and Southern bay scallop, these crosses should be considered as “backcross-like”. The CC5 and CC10 families were raised under the same culture condition following the protocols described by Zheng et al. [31]. Briefly, fertilized eggs of each family were raised separately in a 40 L polyethylene tank for hatching at 22–23°C with a salinity of 30–32‰. Stocking density was kept at 10 individuals/mL by adjusting seawater volume during larval culture. Spats were kept in large concrete tanks until they reached 500∼600 µm. Thereafter, scallops were placed in polyethylene bags and transferred to an outdoor nursery pond. Seventy-day-old juveniles were dispersed into lantern nets (10 layers per net) and hung on a long-line system for grow-out. The densities were adjusted monthly, from 200 juveniles per layer at an early stage to 30 adults per layer after about day 130. At the age of eight months, the offspring (*N* = 94 for each family) were sampled and their shell length (SL), shell height (SH), shell width (SW) and total weight (TW) were measured and recorded. The adductor muscles were dissected and stored in 80% ethanol. The shell color was determined according to the color system developed by Elek and Adamkewicz [32] in this study. As previous studies indicated that shell color in the bay scallop is under single-gene control where white color is recessive [33], [34], the genotypes for orange-colored and white-colored parent were recorded as heterozygote and homozygote respectively.

### Genotyping procedure

DNA from each individual of the two mapping families was extracted from 100 mg of adductor muscle using the traditional phenol/chloroform method. A total of 268 microsatellite markers evaluated and screened in this study were as follows: eight from Roberts et al. [35], 20 suffixed with *AIMS* from Zhan et al. [36], [37]; 28 from Wang et al. [38] and 212 from Li et al. [39]–[42]. Amplification of microsatellite DNA sequences by polymerase chain reaction (PCR) was performed in a 15 µL reaction system containing approximately 50 ng template DNA, 1×PCR buffer, 1.5 mM MgCl_2_, 200 µM dNTPs, 5 pmol of each primer and 0.5 U *Taq* DNA polymerase (Promega). PCR reaction profile was set as follows: denaturation for 5 min at 95°C, followed by 35 cycles of 30 s at 94°C, 30 s at the optimal primer-specific annealing temperature and 30 s at 72°C, with a final extension step at 72°C for 10 min. PCR products were separated using 12% non-denaturing polyacrylamide on a vertical PAGE system AE-6220 (ATTO, Japan). Gels were run at 300 V for 2–3 h depending on amplified fragment sizes, stained with ethidium bromide (EB) and visualized under ultraviolet light.

### Map construction and integration

Polymorphic microsatellite markers derived from each parent were scored as co-dominant markers and a letter (A, B, C or D) was assigned for each allele. Prior to linkage analysis, segregation of alleles was examined for goodness-of-fit to the expected segregation ratio using the chi-square test at α = 0.05 level of confidence. The significant criteria were adjusted for multiple comparisons based on the number of linkage groups (LGs) [43] using the sequential Bonferroni correction [44]. Segregating markers were categorized into four expected segregation types (including null-alleles): 1∶1∶1∶1 type (♀×♂: AB×CD and AB×AC), 1∶2∶1 type (AB×AB), 1∶1 ♀ type (AB×AA or CC), and 1∶1 ♂ type (AA or CC×AB). Informative markers including shell color were grouped into separate sets corresponding to each parent and analyzed independently to construct separate sex-specific framework maps using the double pseudotestcross mapping strategy. The software JoinMap 3.0 [45] was used for map construction from each mapping family with the cross-pollinating (CP) population type, which represents a population resulting from a cross between two heterogeneous parents with linkage phases unknown. Clustering of markers was performed by calculating logarithm of odds (LOD) scores for recombination rate (θ) based on the G^2^ statistic test, which is not affected by segregation distortion [46], thus leading to less incidence of spurious linkage. A LOD threshold >3.5 and a recombination rate threshold <0.4 were set to obtain maps from each mapping population. After assigning the markers into respective LG, heterogeneity in recombination rate for each pair of markers was tested between sexes and families using a G test, which compared the observed numbers of recombinants and non-recombinants with the mean expected numbers.

After creating the individual linkage maps, LGs with common markers on individual maps were merged to create a composite map using “map integration” function of JoinMap. If individual maps produced no significant different recombination rate for all combinations of orthologous markers in certain groups, the integrated LGs were constructed using a LOD weighted average of recombination rate values. When marker order in certain LGs was disturbed between individuals, one parental LG was fixed as a framework and “fix order” command was used to identify the most likely marker order. For assessing the differences in recombination rate between sexes, comparisons of recombination differences between both parents in two mapping families were performed by analyzing all pairwise marker combinations using a two-way contingency G-test as implemented in JoinMap. Graphics of the LGs were constructed using MapChart 2.1 [47].

### Map length and coverage

On the basis of the consensus linkage map, two methods were used to estimate the expected genome length. First, we calculated the average spacing *s* between markers by dividing the total observed map length of all LGs by the number of marker intervals (number of markers minus number of LGs). We estimated genome length (*G_e1_*) by adding 2 *s* to the length of each LG to account for terminal chromosome regions [48]. Second, we calculated estimated genome length (*G_e2_*) by multiplying the length of each LG by (m+1)/(m−1), where m is the number of framework markers on each LG (method 4 in [49]). The average of the two estimates was used as the estimated genome length (*G_e_*) to describe the genome length. The observed genome length (*G_o_*) was calculated as the total length considering all markers. The genome coverage was determined by *G_o_*/*G_e_*.

### QTL detection

Mean value, standard deviations, and the Pearson's correlation analysis of four size-related traits including shell length (SL), shell height (SH), shell width (SW) and total weight (TW) were analyzed with SPSS 12.0. QTL detection was performed by MapQTL 5.0 [50]. Interval mapping (IM) and multiple QTL model (MQM) mapping were utilized to detect any significance associating size-related traits and marker loci. The LOD score significance thresholds were calculated by permutation tests in MapQTL at genome-wide (significant linkage, α<0.05, n = 1000) and chromosome-wide significant level (suggestive linkage, α<0.05, n = 1000). Interval mapping was performed initially using the default parameters of MapQTL. Map intervals exceeding the linkage-group-wide threshold from interval mapping were subsequently selected as cofactors for the MQM procedure. Kruskal-Wallis (KW) analysis (a single marker analysis) was also tested based on one-way analysis of variance (ANOVA), using four parental allelic combinations (m1f1, m1f2, m2f1, m2f2) of markers nearest each QTL peak. Association analysis was carried out to determine the QTL allele substitution effects.

## Results

### Marker segregation

Of a total of 268 microsatellite markers and one phenotypic marker (shell color) screened, 169 markers were informative in at least one family. The number of segregating markers was essentially similar between the two families, with 137 in family CC5 and 119 in family CC10. Within family CC5, 96 and 95 markers segregated in the female and male parents respectively, and 54 markers exhibited a segregation ratio of 1∶1∶1∶1, providing the most valuable co-dominant mapping information. For family CC10, the female and male parent contained 75 and 97 markers respectively, and 53 markers were informative in both parents. Among these markers, 40 were commonly shared with polymorphism across all the parents, and thus allowed integration of two individual genetic linkage maps as allelic bridges.

Null-allele segregation, without which unexpected offspring genotypes could not be explained, was estimated to be 25 loci (25/137, 18.2%) in family CC5 and 22 loci (22/119, 18.5%) in family CC10. Null alleles are not amplified by RCR, and may cause genotyping errors of heterozygote deficiency in population genetic studies [51]. However, null alleles are not a problem for mapping in pedigreed populations [52]. In family-segregation analyses, null alleles can be regarded as recessive alleles for mapping purpose [53]. Thus, we included the informative null-allele segregation for map construction.

Chi-square tests were used to compare all observed progeny ratios of each marker against the genotypic ratios expected under a 1∶1 Mendelian segregation ratio. In family CC5, distorted segregation was observed at a total of 21 loci (15.3%), including 5 loci segregating in the female and 16 loci in the male parent (*P*<0.05). In family CC10, a total of 23 loci (19.3%), including 7 loci in the female and 16 loci in the male parent, showed significant segregation distortion (*P*<0.05). The distribution of distorted markers was asymmetrical between the two sexes, with higher incidence of segregation distortion in male parents than female. Deviations from Mendelian expectations implicated several homologous loci and LGs across families. It is noteworthy that four markers (*AiAD154, AiAD040*, *AiDD105* and *AiSD274*) linked to each other on the homologous group between the two families. Apart from these observations, linked skewed markers appeared scattered across different LGs between families. After sequential Bonferroni corrections, only five deviations showed significant segregation distortions in two mapping families.

### Individual-based linkage maps

Based on the result of linkage grouping at LOD = 3.5, we constructed an individual sex-specific linkage map for each parent. In family CC5, the female and male maps included 96 and 95 makers on 16 and 18 LGs respectively. The female map covered 739.1 cM, with an average interval of 9.2 cM and the number of markers varied from 2 to 13 with an average of 6 markers per LG. The male map covered 696.0 cM, with an average interval of 9.0 cM. The number of loci per LG varied from 2 to 11 with a mean of 5.3 markers. In family CC10, the female and male maps had 75 and 97 markers on 17 and 16 LGs respectively. These two linkage maps covered 657.1 cM and 641.8 cM with an average of 4.4 and 6.0 markers per LG and an averaging spacing of 11.3 cM and 7.9 cM. The details of individual linkage maps, including length, number of markers, marker spacing and largest interval are shown in Table S1.

### Integrated map

**Figure 2:**
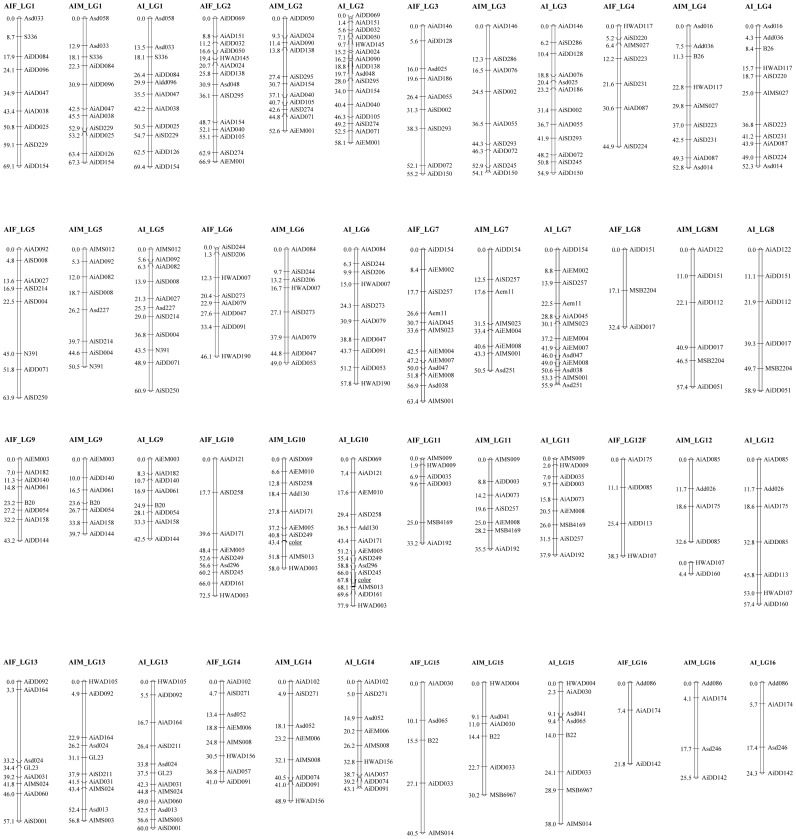
Consensus microsatellite-based linkage map for bay scallop (*Argopecten irradians*) constructed using two hybrid backcross-like families. The female linkage group (left) is named as “AiF_LG”; the male linkage group (middle) is named as “AiM_LG”; the sex-averaged linkage group (right) is named as “Ai_LG”. The phenotypic maker (shell color) placed on LG 10 was underlined.

Bridge markers or homologous loci were used to identify co-linear regions between different individual maps. As shown in Figure 2, the integrated map contained 161 markers distributed over 16 LGs, which was consistent with the haploid chromosome number of the bay scallop [54]. This map covered 849.3 cM with an average inter-marker spacing of 5.9 cM (Table 1). The length of LGs ranged from 24.3 cM to 77.9 cM and the number of markers varied from 4 to 16 per LG (Table 1). The marker for the phenotype of shell color was located on LG10, which was flanked by two microsatellite markers *AiSD245* and *AIMS013*, with the nearest genetic distance of 0.5 cM.

**Table 1:** Number of markers and genetic length for sex-averaged linkage map of bay scallop.

LG	Length of LG (cM)	No. markers	Averaged spacing(cM)
1	69.4	11	6.9
2	58.1	16	3.9
3	54.9	12	5.0
4	52.3	11	5.2
5	60.9	11	6.1
6	57.8	10	6.4
7	55.9	13	4.7
8	58.9	6	11.8
9	42.5	8	6.1
10	77.9	14	6.0
11	37.9	9	4.7
12	57.4	7	9.6
13	60.0	12	5.5
14	43.1	9	5.4
15	38.0	8	5.4
16	24.3	4	8.1
Total/average	849.3	161	5.9
Genome coverage	81.3%		

The genome length of the integrated map estimated by two methods was 1038.1 cM and 1050.7 cM, respectively, with an average of 1044.4 cM. Based on the observed length of the frame map (849.3 cM) and the average expected genome length (1044.4 cM), the percentage of the genome covered by the integrated map was 81.3%.

### Differences in recombination rate between sexes and families

The availability of the common microsatellite markers in the different individual maps allowed for a comparative analysis of recombination rate between sexes and families. Although slight difference in length was observed between female and male maps within the two families (Table S1), a higher was observed in female parents when only common informative markers between sexes were considered. The overall female-to-male recombination rate was 1.13∶1 across all linked markers that are common to both parents in two families. There were 141 and 178 comparable marker pairs in the “between-sexes within each family” category and “within-sexes between families” category, respectively. Of 141 tests of heterogeneity of recombination rate between sexes, 32 (22.7%) were significant at α = 0.05 level using G test. The proportion of significant difference in recombination rate was 20.2% (36/178). When the percentage of marker pairs with significant heterogeneity in recombination rate was compared between these two categories, there was no significant difference (Mann–Whitney test, *P*>0.05).

### QTL mapping

Descriptive statistics of the trait distributions were presented in Table S2. The Kolmogorov–Smirnov test indicated that the variances of SW and TW in the family CC5, and SW, SH and TW in family CC10 were coordinate with normal distribution, however, SL and SW in the family CC5, and SL in the family CC10 were not normally distributed. Phenotypic correlations estimated between any two traits were significant (*P*<0.01), with the Pearson correlation coefficients ranging from 0.772 to 0.941.

**Figure 3:**
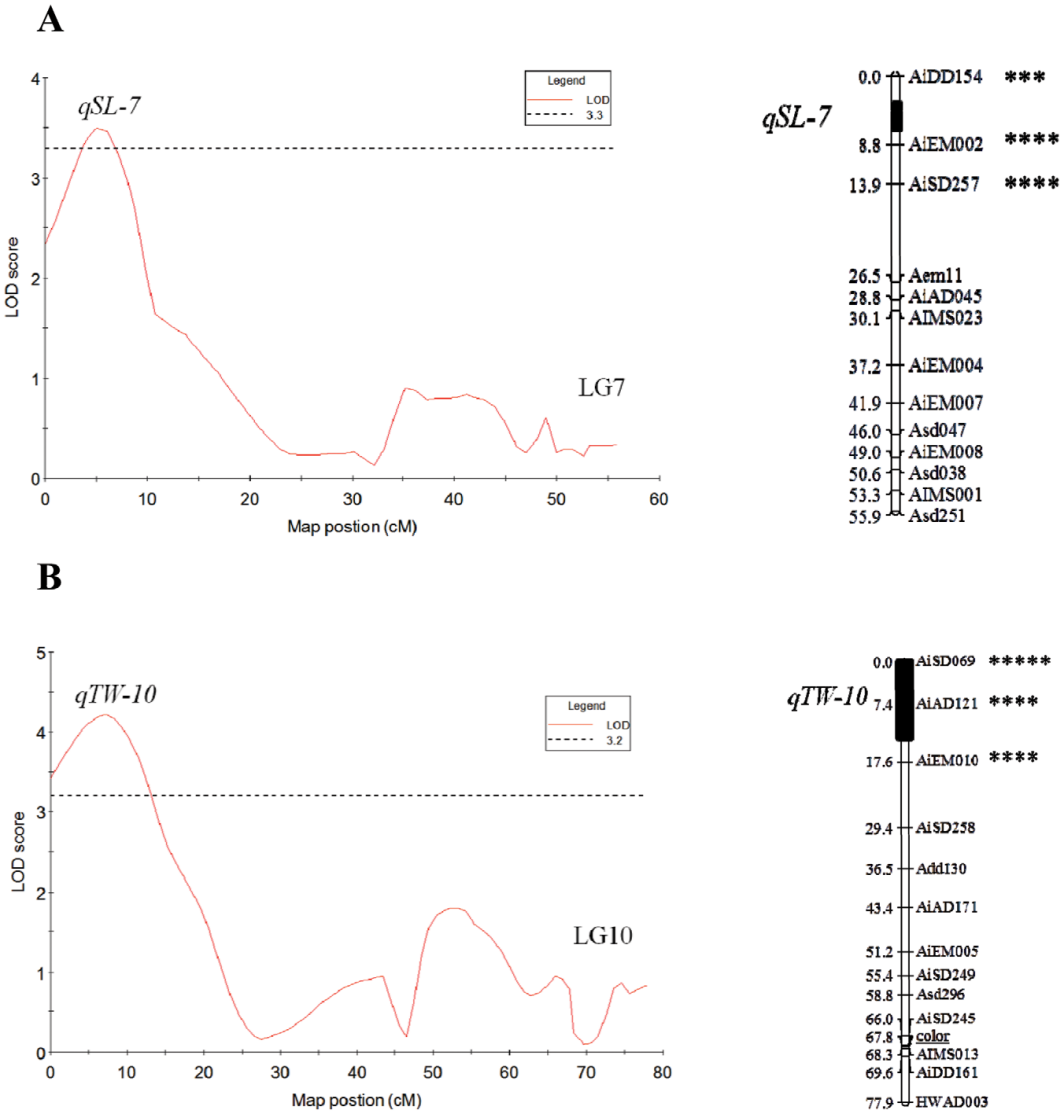
Mapping significant size-related QTL. A, QTL for shell length on LG7; B, QTL for total weight on LG10. QTL were detected with genome-wide threshold. The QTL names are on the left of each box-bar. The significance of Kruskal-Wallis analyses are shown at the right side of linkage group (**P<0.05, *** *P*<0.01, ****P<0.005, ***** *P*<0.001).

QTL affecting four size-related traits, including SL, SH, SW and TW were scanned on a genome scale. Permutation tests using MapQTL indicated that the genome-wide LOD significance thresholds were 3.1–3.3 for SL, SH, SW and TW, whereas the chromosome-wide LOD significance thresholds varied from 2.4 to 2.6. Three significant (genome-wide) and six suggestive (linkage-group-wide) QTL were detected on 6 LGs (LG5, LG6, LG7, LG10 and LG13) in the two mapping populations using the MQM method (Table 2 and Figure 3). The three significant QTL (*qSH-7*, *qSW-10* and *qTW-10*) controlling shell height, shell width and total weight were located on LG7 and LG10, explaining 15.8%, 20.5% and 27.7% of the observed phenotypic variance, respectively. *qSW-10* and *qTW-10* distributed on the same region near marker *AiAD121*. Six suggestive QTL were detected on four different LGs. These QTL explained relatively low phenotypic variance, ranging from 8.9% to 15.3%. Considering QTL positions and confidence intervals, some QTL for SL, SH, SW and TW (*qSL-7* and *qSH-7*, *qSW-10* and *qTW-10*, *qSL-13* and *qTW-13*) were mapped to the same locations of LG7, LG10 and LG13, whereas other QTL were mapped on different LGs. In both CC5 and CC10 families, common QTL were identified on LG10.

**Table 2:** Size-related QTL identified in two families (CC5 and CC10) of bay scallop.

Trait	QTL name	LG	Position (cM)	Confidence interval (cM)#	Nearest marker	LOD	PVE(%)	Phenotype means				Significance
								f1m1	f2m1	f1m2	f2m2	
CC5												
SL	*qSL-7*	7	6.8	4.0–8.2	*AiDD154*	2.9†	15.3	35.4	34.9	38.8	37.5	***
SH	*qSH-7*	7	2.5	4.0–7.0	*AiEM002*	3.1‡	15.8	34.9	37.7	34.9	37.7	****
SW	*qSW-10*	10	5.0	0–10.1	*AiAD121*	3.6‡	20.5	15.5	17.0	15.5	17.0	****
TW	*qTW-5*	5	45.8	43.3–47.6	*N391*	2.7†	9.8	38.0	39.7	35.2	36.1	**
	*qTW-10*	10	7.4	0–13.3	*AiAD121*	4.2‡	27.7	8.2	8.2	10.7	10.7	****
CC10												
SL	*qSL-13*	13	55.3	52.5–57.3	*AIMS003*	2.8†	10.2	35.9	35.9	38.8	38.8	***
SH	*qSH-10*	10	27.6	25.3–30.6	*AIMS013*	2.7†	9.5	34.9	34.7	36.9	36.8	**
TW	*qTW-6*	6	32.4	31.5–33.4	*AiAD079*	2.6†	8.9	8.4	8.6	8.9	10.8	
	*qTW-13*	13	53.1	52.1–53.9	*Asd013*	2.6†	9.6	8.5	8.5	10.0	10.0	*

SL, shell length; SH, shell height; TW, total weight; PVE, percentage of phenotypic variance explained by the QTL; association between four parental genotypes (f1m1, f2m1, f1m2, f2m2) of markers nearest each QTL peak and mean phenotypic values of each trait was tested by Kruskal-Wallis analysis.

# Confidence interval at 95% significance;

† linkage-group-wide;

‡ genome wide;

*P<0.1, **P<0.05, *** *P*<0.01, ****P<0.005, ***** *P*<0.001.

KW test was used for single marker to test associations between phenotypic traits and genotypes of marker nearing each QTL (Table 2). Except for *qTW-6*, all significant and suggestive QTL showed significance by KW analysis. Even though *qTW-6*-nearest maker *AiAD079* showed no significance by KW analysis, other loci flanking this QTL showed significance. In several cases, the associated maker was not always the nearest-QTL one.

## Discussion

As with most other marine invertebrates, mapping resources such as purebred lines, near-isogenic lines and inbred lines were not available in *A. irradians* owing to the biological characteristics including one-year life cycle, high early mortality and inbreeding depression. Since *A. irradians* contains high levels of polymorphisms among local populations [55], we applied an F_1_ pseudo-testcross mapping strategy to take advantages of parental heterozygosity [56]. Considering the co-dominance and easy transferability of microsatellites, two families were used for linkage map construction, which may increase the possibility of placing more microsatellite markers on a consensus map. In addition, the availability of orthologous markers also allowed for a direct comparison of recombination rates and gene orders among different parents. Although moderately dense linkage maps have been previously reported for bay scallop using AFLPs [16], [17], this integrated linkage map based on microsatellite DNA markers represents an important development for marine invertebrate genome research. When compared with the AFLP-based maps, the microsatellite-based linkage map has the advantage of high portability to other families or strains, which may allow more loci to be placed on a genome-wide anchored microsatellite map. Therefore, this study focused on obtaining a general order and distance among these markers rather than the fine resolution of order and distance. The microsatellite-based map may provide a good resource from which markers may be selected for future mapping projects in bay scallop and for comparative studies among other scallops.

Deviations from Mendelian segregation ratio have been detected in previous efforts to construct genetic linkage maps for marine bivalves [9], [15]. For relative scarce linkage maps based on microsatellites, distorted segregation after sequential Bonferroni correction accounted for 30.45% and 29.96% of loci in two mapping families of Zhikong scallop [18]. In this study, the frequency of distorted marker was 15.3% in family CC5 and 19.3% in family CC10. A similar result was also observed in the construction of linkage maps using AFLP markers for bay scallop (14.5% segregation distortion [16]). The level of segregation distortion varies greatly among species and likely reflects the unique characteristics of a genome. Many factors may give rise to segregation distortion. In the Pacific oyster, this phenomenon was explained by a large load of recessive deleterious mutations and low survival of identical-by-descent homozygotes, which was probably caused by deleterious recessive lethal genes [9]. There existed evidence that selective mortality mainly occurred around the time of metamorphosis from the larval to the juvenile stage [57]. High genetic load was confirmed by Hubert and Hedgecock [58], who constructed a Pacific oyster linkage map using 11-day-old larvae, which proved to be effective in reducing segregation distortion. In addition, segregation distortion may result from other factors, such as close linkage of markers to genes or chromosomal regions affecting gametogenesis, competition among gametes for preferential fertilization, genetic drift and chromosomal rearrangements [59], [60]. In this study, it is noteworthy that the proportion of distorted markers was asymmetrical between the sexes, with more occurred in male (hybrid) parents. This result suggested the hypothesis that segregation distortion resulted from heterospecific interaction between genomes of two subspecies of bay scallop and semi-lethal effects may be more evident in hybrid individuals [59], [61]. The masked recessive lethal genes in hybrid parents should be unveiled in backcrossing, which makes more segregation distortions observed in hybrid parents.

Great diversity in shell color has been observed in many marine mollusks, and the variations give considerable implications to breeders since they are associated with differences in survival, growth and other adaptive characteristics [62], [63]. Previous studies indicated that variations in shell color were influenced by environmental factors [62], [64], but in many cases they were genetically determined. In this study, shell color inheritance pattern in CC5 and CC10 families showed a simple 1∶1 segregation, suggesting that shell color in bay scallop is under genetic control. This result is in agreement with other mollusks, such as the mussel *M. edulis* [65], the Pacific abalone *Haliotis discus* [66] and the Zhikong scallop *C. farreri* [18], lending support to the single-gene two-allele model for genetic control of shell color. The fact that white× (white×orange) crosses produced about 50% white and 50% orange progeny implies that orange parent is heterozygous and white allele is recessive to orange. In our previous study, we have detected an association between orange color and higher quantitative traits in larval and adult stages of bay scallop [29]. The microsatellite markers linked to shell color obtained in this study provide an opportunity to breed colorful lines with enhanced growth performance for bay scallop.

It is of interest to examine the difference of recombination rate between sexes and individuals for any species. Significant difference in sex recombination ratios has been reported in many aquatic animals [67]. In this study, the recombination rate in female parents was higher than the males, with an overall female-to-male ratio of 1.13∶1. This is consistent with higher female recombination rates and longer map lengths observed in other marine mollusks, such as the oyster (*Crassostrea gigas*, [9], [58]) and the abalone (*Haliotis rubra*, F∶M = 1.45∶1 [68]). Difference in recombination rate between sexes may result from various complex factors [69]. Since bay scallops are functional hermaphrodites that exhibit both male and female reproductive functions in a single individual, it should be possible to observe recombination in the same individual acting as a male or a female parent. Further investigation is needed to understand the sex specificity in recombination rate of the hermaphroditic bay scallop.

Family-specific recombination rates were also demonstrated in linkage maps based on multiple mapping populations. Significant differences in recombination and orders of marker were evident among same-sex parents of different families in *C. gigas*, which suggested that polymorphism for chromosomal rearrangements may exist in natural populations [58]. Similarly, Zhan *et al.* [18] attributed the heterogeneity in recombination rate to individual difference, not sexes. In this study, there was no significant difference in the percentage of marker pairs with significant heterogeneity in recombination rate between “within-sexes between families” and “between-sexes within each family” category, which suggests that heterogeneities in recombination rates would be mainly due to individual differences. Although chromosomal rearrangements [58], sampling variations associated with sample sizes and statistical treatments [70], and environmental differences between mapping families [71] may be possible causes for this common phenomenon, the underlying mechanism is still not well understood.

Accurate mapping techniques have been developed in recent years to detect QTL for economically important traits. The efficiency for QTL mapping depends on heritability of traits, average allelic substitution effect of the alleles involved, recombination distance between the QTL and associated markers, and sample size of the progeny [72]. For most shellfish species, they are broadcast spawners with high fecundity. The large number of progeny produced in a single family can ensure the accuracy of linkage map construction and QTL mapping. In addition, most bivalve mollusks possess high levels of heterozygosity. Hence, pseudo-testcross strategy would be ideal for QTL analysis in marine bivalves [16], [73]. However, QTL mapping study using crosses between heterozygous parents from a breeding population has limited QTL discrimination. As QTL allele-substitution is measured against a heterogeneous background, the detected QTL may be limited to loci with specific genetic configurations and/or strong effects. Moreover, QTL are environmentally influenced and it is difficult to dissect the genetic and environment effects [74], [75]. As a result, QTL-associated markers need to be validated in different genetic backgrounds and environments for application in molecular breeding [76].

In this study, four size-related traits were highly correlated with each other, with the Pearson's correlation coefficient being greater than 0.772. Separate QTL analysis showed that LOD peaks controlling two size-related traits were located on the same region of LG7, LG10 and LG13, indicating either the linkage of two QTL or the presence of a single QTL with pleiotropic effects. Common QTL-underlying phenotypes may be the most likely explanation to the co-location of LOD peaks [70]. However, given the current marker density and unavailability of selected lines, it is rather difficult to distinguish between a single gene (or gene cluster) with individual QTL peak. More markers are needed to identify the full spectrum of genetic components contributing to size differences and to narrow down the distance between a marker and a QTL peak.

Three significant and six suggestive QTL were mapped on five LGs in this study, while in Zhikong scallop, a total of eight size-related QTL were detected on nine different LGs using a genome scan [18]. The proportion of variance explained by the significant QTL varied from 15.8% to 27.7%, which is similar to the results of 6.4–19.2% found in Zhikong scallop [18]. Among the three significant QTL identified in this study, *qSW-10* and *qTW-10* located on LG10, explained high percentage of the observed phenotypic variance, suggesting that there may exist a few QTL/genome regions that control size-related traits of the bay scallop. Our findings agree well with some previous studies in crops and domestic animals that a large proportion of quantitative variation can be explained by the segregation of a few major QTL [76].

In this study, two mapping families produced considerably different results, with only QTL on LG10 shared between them. Similar results were also obtained from the QTL analysis with multiple families in plants and insects. In maize, two independent samples evaluated in four environments were used to detect 107 QTL, of which only 20 were in common [74]. Previous studies indicated that family-specific QTL could be caused by statistical sampling and fixation of some QTL in some families [18], [74]. Since bay scallops are highly fecund animals with intensive natural selection on early life stages, biased survival of progeny may contribute to the different QTL results of different families. Additionally, we used hatchery scallops as mapping parents, which may be fixed for homozygous QTL allele during aquaculture practice. No segregation occurred in mapping parents. This study demonstrates the need of using multiple families for QTL mapping analysis in bay scallop.

A one-way ANOVA revealed that allelic substitution of microsatellite near significant and suggestive QTL showed large effects on size-related traits. The genotypes at these microsatellite loci may be useful for growth improvement through MAS. It could be expected that only the closest marker to the QTL peak showed a significant association, but in several cases, the associated marker was not always the closest one. There could be multiple explanations for this observation, including low mapping information of the closest marker, large genome region in linkage disequilibrium with QTL and possible existence of a secondary segregating QTL [77]. Future studies focusing on genotyping more markers would be valuable for improving the resolution of QTL mapping.

## Conclusions

In this study, a consensus genetic linkage map was constructed for the bay scallop using microsatellite markers, providing a base-line map for this important aquaculture species. It offers a scaffold that can be applied for further genetic studies, such as increasing marker density, QTL analysis of economic traits, genome assembly and addressing the problems of scallop evolution. Size-related QTL analysis was performed in two backcross-like families and nine QTL were detected on five LGs. These results provide a good starting point for fine-mapping of the identified QTL for MAS and eventually identifying candidate genes responsible for growth-related traits for gene-assisted selection (GAS) in the bay scallop.

## Supporting Information

Table S1 **Statistics for individual sex-specific linkage maps of bay scallop (** ***Argopecten irradians*** **) in two reference families CC5 and CC10.**
(DOC)

Table S2 **Statistics of four size-related traits in two mapping families CC5 and CC10 (± standard deviation).**
(DOC)
