# Supplementary material for: A Consensus Microsatellite-Based Linkage Map for the Hermaphroditic Bay Scallop (Argopecten irradians) and Its Application in Size-Related QTL Analysis
Source: PLoS One. 2012 Oct 16;7(10):e46926. doi: 10.1371/journal.pone.0046926 (PMC3473060; doi:10.1371/journal.pone.0046926)
Supplement: Table S1 — Statistics for individual sex-specific linkage maps of bay scallop ( Argopecten irradians ) in two reference families CC5 and CC10. (DOC) [file pone.0046926.s001.doc]

**Table S1.** Statistics for individual sex-specific linkage maps of the bay scallop (*Argopecten irradians*) in two reference families CC5 and CC10.

| CC5 | Female |  |  | Male |  |  |
| --- | --- | --- | --- | --- | --- | --- |
| Group | Length  (cM) | No. markers | Marker spacing (cM) | Length  (cM) | No.  markers | Marker spacing (cM) |
| 1 | 61.1 | 8 | 8.7 | 57.8 | 7 | 9.6 |
| 2 | 70.5 | 13 | 5.9 | 48.7 | 11 | 4.9 |
| 3 | 41.0 | 6 | 8.2 | 50.6 | 5 | 12.7 |
| 4 | 39.4 | 5 | 9.9 | 55.1 | 5 | 13.8 |
| 5 | 55.1 | 6 | 11.0 | 45.4 | 7 | 7.6 |
| 6a | 43.8 | 6 | 8.8 | 8.6 | 2 | 8.6 |
| 6b | - | - | - | 40.2 | 5 | 10.0 |
| 7 | 64.6 | 10 | 7.2 | 49.6 | 5 | 12.4 |
| 8 | 29.6 | 2 | 29.6 | 47.9 | 5 | 12.0 |
| 9 | 33.3 | 7 | 5.6 | 39.6 | 7 | 6.6 |
| 10 | 70.9 | 8 | 10.1 | 84.1 | 9 | 10.5 |
| 11 | 40.7 | 4 | 13.6 | 21.5 | 4 | 7.2 |
| 12 | 24.8 | 3 | 12.4 | 23.2 | 3 | 11.6 |
| 13a | 59.2 | 7 | 9.9 | 46.1 | 5 | 11.5 |
| 13b | - | - | - | 15.4 | 3 | 7.7 |
| 14 | 45.2 | 5 | 11.3 | 39.9 | 6 | 8.0 |
| 15 | 38.7 | 4 | 12.9 | 18.7 | 4 | 6.2 |
| 16 | 21.2 | 2 | 21.2 | 3.6 | 2 | 3.6 |
| Total/average | 739.1/  46.2 | 96/6.0 | -/9.2 | 696.0/  38.7 | 95/5.3 | -/9.0 |
| CC10 | Female |  |  | Male |  |  |
| Group | Length  (cM) | No. markers | Marker spacing (cM) | Length  (cM) | No. markers | Marker spacing (cM) |
| 1 | 37.8 | 4 | 12.6 | 59.5 | 8 | 8.5 |
| 2 | 76.2 | 8 | 10.9 | 50.3 | 8 | 7.2 |
| 3 | 48.7 | 4 | 16.2 | 37.8 | 6 | 7.6 |
| 4 | 36.1 | 4 | 12.0 | 50.5 | 6 | 10.1 |
| 5 | 38.7 | 5 | 9.7 | 43.5 | 5 | 10.9 |
| 6 | 50.4 | 4 | 16.8 | 49.3 | 8 | 7.0 |
| 7 | 57.4 | 7 | 9.6 | 46.0 | 6 | 9.2 |
| 8 | 36.8 | 3 | 18.4 | 34.4 | 4 | 11.5 |
| 9 | 57.0 | 5 | 14.3 | 38.7 | 6 | 7.4 |
| 10 | 49.4 | 6 | 9.9 | 46.0 | 10 | 5.1 |
| 11 | 38.5 | 4 | 12.8 | 25.0 | 5 | 6.3 |
| 12 | 29.6 | 3 | 14.8 | 32.6 | 4 | 10.9 |
| 13a | 1.1 | 2 | 1.1 | 45.1 | 7 | 7.5 |
| 13b | 7.5 | 3 | 3.8 | - | - | - |
| 14 | 36.9 | 5 | 9.2 | 35.8 | 7 | 6.0 |
| 15 | 29.2 | 4 | 9.7 | 25.5 | 4 | 8.5 |
| 16 | 25.8 | 4 | 8.6 | 21.8 | 3 | 10.9 |
| Total/average | 657.1/  38.7 | 75 | -/11.2 | 641.8/  40.1 | 97 | -/8.4 |
