# Supplementary material for: A Consensus Microsatellite-Based Linkage Map for the Hermaphroditic Bay Scallop (Argopecten irradians) and Its Application in Size-Related QTL Analysis
Source: PLoS One. 2012 Oct 16;7(10):e46926. doi: 10.1371/journal.pone.0046926 (PMC3473060; doi:10.1371/journal.pone.0046926)
Supplement: Table S2 — Statistics of four size-related traits in two mapping families CC5 and CC10 (± standard deviation). (DOC) [file pone.0046926.s002.doc]

**Table S2.** Statistics of four size-related traits in two mapping families CC5 and CC10 (± standard deviation).

| Family | Shell length (mm) | Shell height (mm) | Shell width (mm) | Total weight (g) |
| --- | --- | --- | --- | --- |
| CC5 | 37.40 ± 4.12 | 36.08 ± 3.99 | 16.09 ± 2.02 | 9.14 ± 2.93 |
| CC10 | 36.61 ± 4.33 | 35.27 ± 4.06 | 15.34 ± 2.30 | 8.21 ± 2.70 |
